# *Cepaea* spp. as a source of *Brachylaima mesostoma* (Digenea: Brachylaimidae) and *Brachylecithum* sp. (Digenea: Dicrocoeliidae) larvae in Poland

**DOI:** 10.1007/s00436-019-06516-2

**Published:** 2019-11-25

**Authors:** Elżbieta Żbikowska, Anna Marszewska, Anna Cichy, Julita Templin, Anna Smorąg, Tomasz Strzała

**Affiliations:** 1 Department of Invertebrate Zoology and Parasitology, Faculty of Biology and Environment Protection, Nicolaus Copernicus University in Toruń, Toruń, Poland; 2 Department of Genetics, Faculty of Biology and Animal Science, Wrocław University of Environmental and Life Sciences, Wrocław, Poland

**Keywords:** *Brachylaima mesostoma*, *Cepaea hortensis*, *Cepaea nemoralis*, *Brachylecithum* sp., Prevalence, Shell morph

## Abstract

Parasite diagnostics were carried out on 11 Polish populations of *Cepaea* spp. In three of them, coming from the roadside ditches of a village (Rytel, northern Poland), very high (up to 60%) prevalence of *Brachylaima mesostoma* was observed. This study provides the first molecular evidence of the presence of *B. mesostoma* inside *Cepaea* spp. in Europe. In a few snails from a population found in a private garden in a small town (Chełmża, northern Poland), larvae of *Brachylecithum* sp. were present. Cercariae and/or metacercariae of *B. mesostoma* were observed in both species of *Cepaea*: *C. hortensis* and *C. nemoralis*, whereas larvae of *Brachylecithum* sp. were found only in *C. nemoralis*. Both species of parasites inhabited snail hepatopancreas whose structure was significantly damaged by larvae. There was no significant connection between parasite invasion and snail host morphotype. The research did not allow the reasons for the high prevalence of *B. mesostoma* in *Cepaea* spp. to be explained, and also did not explicitly indicate how the parasite invaded *Cepaea* spp. individuals making them, at the same time a second intermediate host. However, it poses important questions about the life cycle of the parasite that may threaten extensively kept small-size farms of poultry.

**Electronic supplementary material:**

The online version of this article (10.1007/s00436-019-06516-2) contains supplementary material, which is available to authorized users.

## Introduction

Transmission of Digenea (Platyhelminthes: Trematoda) to hosts in terrestrial life cycles as a rule takes place in a passive way. Because of this limitation, the parasites have developed some strategies for completing life cycles (Niewiadomska and Pojmanska 2011). One of the digenean family characterized by a terrestrial life cycle is Brachylaimidae Joyeux & Foley, 1930, which includes the large superfamily Brachylaimoidea Allison, 1943 (Hildebrand et al. 2016). One of its genus—*Brachylaima* Dujardin, 1843 (Platyhelminthes: Digenea)—includes more than 70 species (Reda and El-Shabasy 2016). The presence of the adult parasites inside birds and mammals has been noted on almost all continents (Butcher and Grove 2005; Fedatto-Bernardon et al. 2017; Liatis et al. 2017; Suleman and Khan 2016), including Europe (e.g. in Poland) (Okulewicz 2014). Terrestrial snails play the role of the first and the second intermediate hosts for *Brachylaima* sp. Intermediate hosts of flukes of the genus *Brachylaima* include land snails belonging to different families (Cribb 1990; Cribb and O’Callaghan 1992; Stenko and Stenko 1988; Thiengo and Amato 1995). Invasive metacercariae are transmitted trophically to the final host. Intensified study on the genus *Brachylaima* was carried out due to its medical significance and the low specificity of parasites to host species at all levels of the life cycle (Butcher and Grove 2003; Cribb 1990; Segade et al. 2011; Pavlov 1946; Stenko and Stenko 1988). Another species of trematodes belonging to the family Dicrocoeliidae Looss, 1899 have long-tailed xiphidocercariae which have emerged from terrestrial snails in, so-called, mucoid balls which support their survival in the environment. The life cycle of these parasites consists of three or even four hosts, including the paratenic host (Niewiadomska and Pojmanska 2011). One of the largest dicrocoeliine genera is *Brachylecithum* Shtrom, 1940. The knowledge of hosts of *Brachylecithum* is still incomplete. The first intermediate hosts of the parasite may be the common genus *Cepaea* Held, 1838. Their second intermediate hosts are some arthropods, while their definitive hosts include mostly birds (Hildebrand et al. 2016).

This feature of the parasites as well as their potential and real medical and veterinary significance were the main reasons for our research on *Cepaea nemoralis* (Linnaeus, 1758) and *C. hortensis* (Müller, 1774), which belong to the Helicidae family. Both species of *Cepaea* are widely spread in Europe and have a distinctive shell polymorphism which has been the subject of many studies (e.g. Cameron and Cook 2012). The snails were listed as hosts of parasitic nematodes (Grewal et al. 2003; Morand 1989) as well as of one digenetic trematode—*Brachylecithum* sp.—first described in Poland (Hildebrand et al. 2016), both transmitted trophically. Visually hunting predators (birds and/or mammals) have been documented as a very strong factor affecting *Cepaea* sp. morph frequency (Cook 1998; Ożgo 2009).

In the presented research, we aimed to extend the knowledge on the presence of Digenea in the common species of *Cepaea*. Due to the scarcity of studies about the presence of parasites in this genus, we expected that the real infection of *Cepaea* spp. is higher than the one indicated by researchers so far. The digenetic trematodes heavily exploit the hepatopancreas of the first intermediate hosts; therefore, we supposed that this organ of infected individuals of *Cepaea* spp. will be characterized by heavy devastation. Considering that visually hunting predators play the role of the final hosts of parasites developing in *Cepaea*, it was hypothesised that there is a connection between parasitic invasion and snail morphotypes.

## Material and methods

### Field sampling

**Fig. 1:**
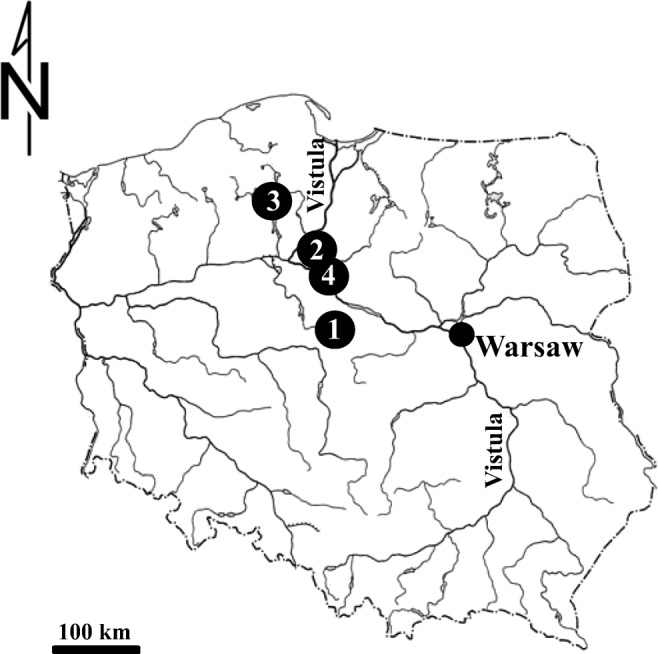
Study sites: 1—Bytoń, 2—Chełmża, 3—Rytel, 4—Toruń

Mature specimens of *Cepaea* spp. were sampled from April to May in 2016. They were collected from 11 research areas of central and northern Poland in four different localities: Bytoń (I.—52° 27′ 17.28″ N, 18° 26′ 23.28″ E), Chełmża (I.—53° 11′ 20.76″ N, 18° 37′ 24.599″ E; II.—53° 11′ 0.6″ N, 18° 36′ 14.399″ E), Rytel (I.—53° 44′ 56.76″ N, 17° 46′ 17.399″ E; II.—53° 44′ 49.56″ N, 17° 46′ 9.48″ E; III.—53° 44′ 57.48″, 17° 46′ 24.239″ E), Toruń (I.—53° 1′ 16.68″ N, 18° 34′ 5.16″ E; II.—53° 1′ 4.44″ N, 18° 34′ 41.519″ E; III.—53° 1′ 4.08″ N, 18° 35′ 34.44″ E; IV.—53° 0′ 11.16″ N, 18° 20′ 38.4″ E; V.—53° 1′ 38.64″ N, 18° 35′ 59.279″ E) (Fig. 1).

### Examination of snails and digenean larvae

Wąsowski and Penkowski’s (2003) key was used for morphological identification of snails to species. Types of changes in shell morph frequencies were identified in accordance with Ożgo et al. (2017). Morphotypes were divided into shell ground colour [yellow (Y), pink (P), and brown (B)] and changes in banding categories [mid-banded (00300), three-banded (00345), five-banded (12345)], whereas bands joined together are written in brackets [()].

All snails were dissected and deprived of removing their shell. Using a light microscope (Primostar Carl Zeiss), the internal organs, such as gonad, digestive gland, and hepatopancreas, were checked for the presence of parasites. Morphological identification of parasites was performed according to available descriptions and diagnostic pictures (Hildebrand et al. 2016; Köse et al. 2015). Pathological changes in snail organs were studied histologically.

### Histological examination

Histological evaluation of damage to snail tissues caused by the presence of parasites was performed by comparing organ sections from infected and non-infected snails using the standard method (Slaoui and Fiette 2011). Organs were removed from snails and immediately fixed in Bouin’s fluid. Following fixation, the samples were dehydrated in a graded ethanol series, cleared with xylene, infiltrated, embedded in paraffin wax, sectioned at 4.5 μm, and stained with haematoxylin and eosin using standard protocols.

### DNA extraction, PCR amplification, sequencing, and phylogenetic analyses

For molecular identification, parasite larvae were isolated from fresh snail tissue and preserved in ethanol (96%) and frozen (at − 20 °C). Prior to DNA extraction, larvae were centrifuged at 5000×*g* for 5 min and washed three times in distilled water. Total genomic DNA was isolated with Sherlock AX (A&A Biotechnology, Gdynia, Poland) according to the producer’s manual. The quality and quantity of the isolated DNA were assessed in gel electrophoresis (1% agarose gel). A fragment of the ribosomal DNA, spanning the sequences of internal transcribed spacers 1, 2, and 5.8S (ITS), was amplified using the forward primer its5Trem (5′-GGAAGTAAAAGTCGTAACAAGG-3′) and the reverse primer its4Trem (5′-TCCTCCGCTTATTGATATGC-3′) (Dvořák et al. 2002) following PCR conditions described by Dvořák et al. (2002). The products obtained were purified with Clean-Up (A&A Biotechnology, Gdynia, Poland) according to the manufacturer’s instructions. DNA product sequencing in both directions was carried out by Genomed S. A., Warsaw, using Sanger method and Applied Biosystems 3730XL DNA analyzer. To reveal the species belonging of the sample, we conducted a phylogeny reconstruction based on the dataset of 44 homological DNA sequences obtained from Genbank (Table SM1). Dataset was first aligned using Muscle algorithm (Edgar 2004) implemented in Seaview (Gouy et al. 2010) and after alignment sequences were cut to obtain proper block of sequences. Next, we chose best-fit substitution model for the dataset, using jModelTest 2.1.10 (Darriba et al. 2012). Finally, to identify species belonging of analysed samples, we used MrBayes 3.2.6 (Ronquist et al. 2012) and PhyML 3.0 (Guindon and Gascuel 2003; Guindon et al. 2010) to construct the phylogenetic tree with Bayesian Inference (BI) and Maximum Likelihood (ML) approach, using GTR +G +I best-fit substitution model. In MrBayes, two randomly started, independent runs (robots) were used (with four Markov chain for each robot). Trees were sampled every 200th generation for 25,000,000 generations (Markov chain steps), to be sure that final consensus tree will consist of trees collected when both runs were already converged—i.e. average standard deviation between runs was much lower than 0.01 for all trees (0.002 was the highest value of SD between both robots). In PhyML, five random starting trees and SPR tree improvement were used along with boostrap analysis (1000 replications) to test tree topology.

## Results

**Fig. 2:**
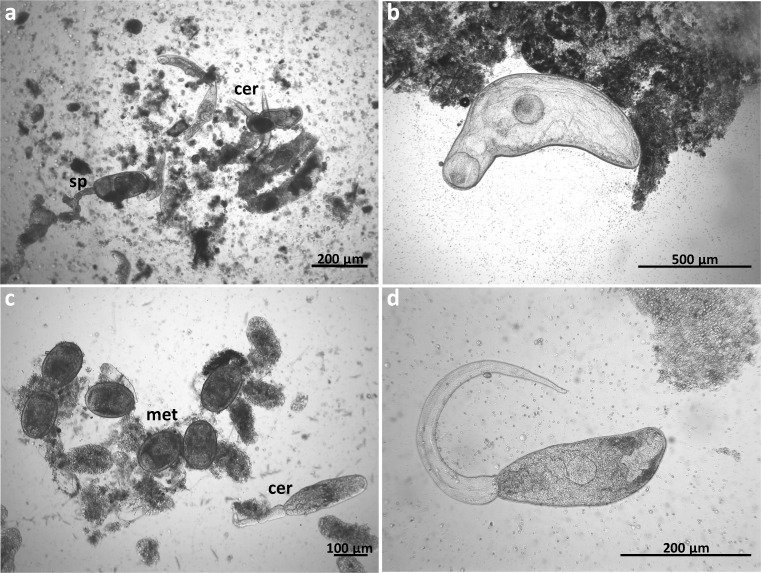
Recorded Digenea: **a** cercariae and sporocysts of *Brachylaima mesostoma*, **b** metacercariae of *Brachylaima mesostoma*, **c** metacercariae of *Brachylecithum* sp., **d** cercariae of *Brachylecithum* sp.

In total, 934 snails were investigated—759 specimens of *C. nemoralis* and 175 of *C. hortensis*. Both species of snails co-exist in almost all research areas (9 out of 11 study fields) (Table 1). Almost 13% of the total collected *Cepaea* spp. were infected with digenean larvae (17.1% of *C. hortensis*, and 11.9% of *C. nemoralis*). The presence of the parasites was observed in only four research areas (all study areas from Rytel (I, II, III) and one area from Chełmża (I)) (Table 1). Almost all recorded parasites, according to morphological diagnostics, were initially classified as *Brachylaima* sp. (Fig. 2) (98.3% of infected *Cepaea* spp.) and *Brachylecithum* sp. (Fig. 2) (a few infected *C. nemoralis*).

**Table 1:** Number of collected *Cepaea* spp. and their infection [%]

Sampling area	*Cepaea nemoralis*		*Cepaea hortensis*	
	No. of collected	% of infected	No. of collected	% of infected
Bytoń I	64	0	0	–
Chełmża I	75	2.67^a^	0	–
Chełmża II	132	0	7	0
Rytel I	49	44.9^b^	15	33.33^b^
Rytel II	41	53.66^b^	20	60^b^
Rytel III	93	47.31^b^	49	26.53^b^
Toruń I	67	0	32	0
Toruń II	77	0	4	0
Toruń III	51	0	7	0
Toruń IV	50	0	27	0
Toruń V	60	0	14	0
Sum	759	11.86	175	17.14

^a^*Brachylecitum* sp.

^b^*Brachylaima mesostoma*

**Fig. 3:**
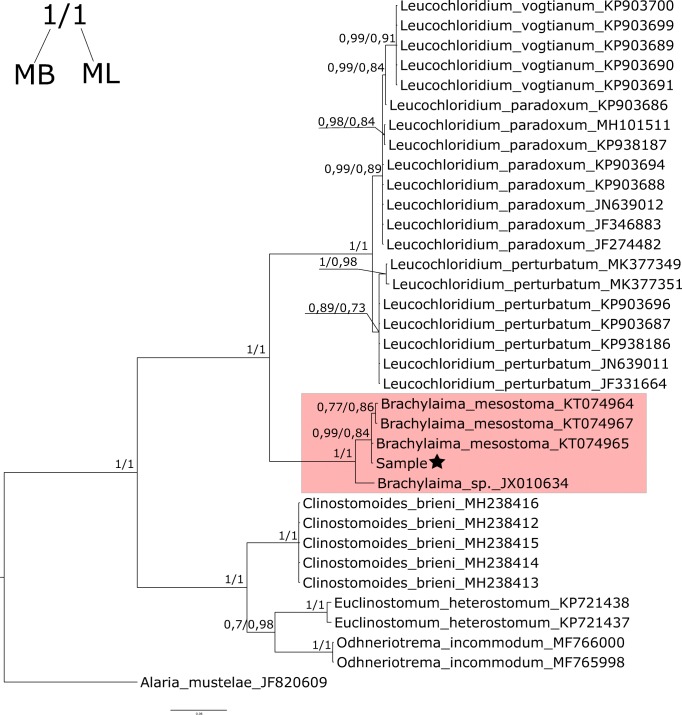
Bayesian phylogenetic tree presenting systematic position of analysed DNA sample (sample is indicated with a star). Sequences of *Alaria mustelae* were used as outgroup for rooting. Numbers along node are posterior probability values and bootstrap value for nodes significance

Molecular identification of parasite species has been successfully carried out for *Brachylaima* sp. All sequenced DNA samples had the same haplotype (Genbank accession number MN218602). Both ML and BI trees had the same topology, on which DNA sequence revealed in this study belonged to *Brachylaima mesostoma* as it creates highly significant (probability 100%) node with other *B. mesostoma* representatives on the created tree (Fig. 3). DNA isolation from the collected specimens of *Brachylecithum* sp. was unsuccessful.

The prevalence of *Brachylaima mesostoma* in snail populations from Rytel area was high and reached over 53% for *C. nemoralis* and 60% for *C. hortensis* (Table 1). *Brachylecithum* sp. was listed only in Chełmża (I)—2.7% of *C. nemoralis* (Table 1).

The infected snails were the hosts of cercariae and/or metacercariae of the parasites. We observed both types of larvae inside all *C. nemoralis* infested with *Brachylecithum* sp. The hosts of *B. mesostoma* were mostly infected with metacercariae (41%), both types of the parasite larvae were found inside 32% of infected snails, while hosts of only cercariae accounted for 27% of infected animals.

**Fig. 4:**
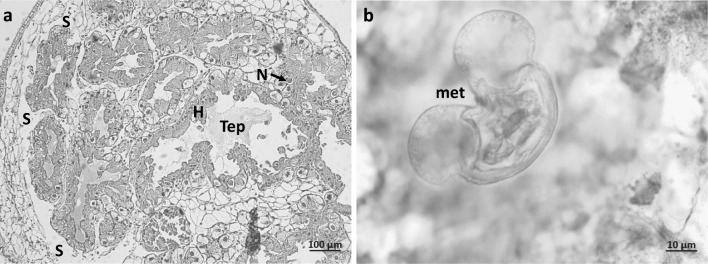
Photomicrographs of hepatopancreas of *Cepaea* sp. **a** naturally infected with metacercariae of *Brachylaima mesostoma* (H—hepatopancreatic tubules separated by connective tissues; Tep—tubule epithelial cells bound the lesion; N—cell nucleus; S—separation of cells), **b** with magnified metacercariae (M—metacercariae of *B. mesostoma*)

Histological sections of infected snails revealed that the nuclear-cytoplasmic ratio in hepatopancreas cells was shifted in favour of the nuclei (large nuclei of the cells of infected individuals, relatively little cytoplasm). Epithelial cells lining the digestive tubules of the infected hepatopancreas had an irregular and flattened shape (Fig. 4, Fig. SM1). As a result, the lumen of the digestive tract was larger than in the non-infected hepatopancreas. Parasitic larvae were visible among the damaged parts of the organ (Fig. 4).

We recorded 26 morphotypes of *Cepaea* spp. The most common shell ground colours were yellow (Y), pink (P), and brown (B), respectively (Table 2), whereas the most frequently presented change in banding categories was mid-banded (00300). From 40.78 to 51.32% of individuals belonging to each category of colours of shell morphotypes from research sites in Rytel (the study areas with the highest infection of snails) were infected (Table 3). However, snails with pink (P) and brown (B) shells were more often infected by metacercariae and metacercariae together with cercariae (72%) than snails with bright yellow shells (69%).

**Table 2:** Morphotypes of collected *Cepaea* spp. from study areas

Morphotypes	% of collected *Cepaea nemoralis*	% of collected *Cepaea hortensis*	% of collected *Cepaea* spp.
Y 00000	2.50	5.14	3
Y 00300	30.96	34.86	31.69
Y 00345	1.84	1.14	1.71
Y 12345	11.33	19.43	12.85
Y 123 (45)	6.19	4.57	5.89
Y (12)345	0.13	0	0.11
Y (12)3(45)	2.24	1.14	2.03
Y (123)(45)	0.13	0	0.11
Y (12345)	0.66	0.57	0.64
P 00000	3.16	10.29	4.5
P 00300	17.13	13.71	16.49
P 00345	1.19	0.57	1.07
P 12345	2.63	2.86	2.68
P 12(345)	1.05	0	0.86
P 123(45)	1.98	0	1.61
P (12)345	0.13	0	0.11
P (12)3(45)	3.43	0	2.78
P (123)(45)	0.26	0	0.21
P (12345)	1.71	0	1.39
B 00000	3.56	4	3.64
B 00300	6.46	1.14	5.46
B 12345	0.26	0	0.21
B 123(45)	0.26	0.57	0.32
B (12)3(45)	0.26	0	0.21
B (123)(45)	0.13	0	0.11
B (12345)	0.39	0	0.32

**Table 3:** Number of morphotypes of collected *Cepaea* spp. and their infection [%] from Rytel

	*Cepaea nemoralis*		*Cepaea hortensis*		% of infected *Cepaea* spp.	% of infected colour of morphotypes
Morphotypes	No. of collected	% of infected	No. of collected	% of infected		
Y 00000	4	50	0	–	50	40.78
Y 00300	66	43.94	31	41.93	43.3	
Y 00456	2	0	0	–	0	
Y 12345	33	45.45	23	17.39	33.93	
Y 123(45)	9	77.78	5	0	50	
Y (12)3(45)	3	66.67	2	50	60	
Y (12345)	0	–	1	–	0	
P 00000	10	60	8	75	66.67	51.32
P 00300	27	44.44	8	37.5	42.86	
P 12345	13	61.54	1	0	57.14	
P 12(345)	1	0	0	–	0	
P 123(45)	3	0	0	–	0	
P (12)3(45)	3	100	0	–	100	
P (123)(45)	1	100	0	–	100	
P (12345)	1	0	0	–	0	
B 00000	2	50	3	33.33	40	50
B 00300	5	40	1	100	50	
B 123(45)	0	–	1	100	100	

## Discussion

Our finding of larval stages of *Brachylaima mesostoma* in *Cepaea hortensis* and *Cepaea nemoralis* is the first molecular evidence in Europe, and indicates that intermediate hosts of this parasite include other species of snails than those described by Stenko and Stenko (1988). However, there are previous reports on the presence of *Brachylaima* spp. in *Cepaea* sp. (Korol 2012). Adult forms of *Brachylaima mesostoma*, a parasite of *Turdus iliacus* (Braun 1902), developed in quail, rabbit (Pavlov 1946), and chickens (Stenko and Stenko 1988) as a result of experimental invasion. The possibility of developing fertile adult forms of this parasite in poultry indicates that *B. mesostoma* can be a threat for breeding birds. The threat concerns particularly free-range poultry production, where *Cepaea* spp.—a source of protein for the chickens—have wide access to bird droppings. On the other hand, the presence of adult *Brachylaima* inside both experimentally and naturally infected mammals (Pavlov 1946; Torres et al. 2003) raises the question about the possible role of synanthropic rodents in the cycle of this parasite, which will be checked in our future study.

The high spring prevalence of *B. mesostoma* in snail populations from Rytel village is of particular interest. The quantitative data indicate a significant source of the parasite eggs inflowing to the biotope inhabited by *Cepaea* sp. According to Stenko and Stenko (1988), the development of larvae of *B. mesostoma* inside chickens lasts about a week, while once infected snails can produce the cercariae for many months. In fact, the examination of chicken faeces in the surrounding farms conducted during summer did not show the invasion of the parasite in the chickens (our unpublished data). However, we postulate that high prevalence of *B. mesostoma* in *Cepaea* sp. could be affected by parasitic invasion in poultry in a view of the results of Stenko and Stenko (1988), who emphasized that the experimental infestation with *B. mesostoma* was fatal to chickens.

Naturally infected snails consisted of three groups: (i) hosts for cercariae, (ii) hosts for metacercariae, and (iii) hosts for both larval stages. The presence of only cercariae or metacercariae may suggest two probable ways of infection of *Cepaea* spp.: (i) by eating the parasite eggs in the final host’s faeces or (ii) by eating the cercariae developing inside the first intermediate host. The transmission of cercariae released from the first to the second intermediate host snails of the same species is widely present in Digenea (Zimmermann et al. 2016). Transmission of the trematodes in the terrestrial environment precludes the possibility of active penetration of emerged cercariae into the second intermediate host. It is well documented that *Cepaea* spp. show cannibalistic behaviour, especially for weaker individuals (Ożgo and Bogucki 2009) which explains the alimentary way of invasion of cercariae from the first to the second intermediate snail hosts. The damage to the hepatopancreas of infected individuals indicates reduced fitness of snails.

The presented high frequency of the mixed invasion (cercariae and metacercariae) may also suggest an alternative way leading to the coexistence of the both larval stages inside one snail. There are well-known cases of species of Digenea whose cercariae in the same host can transform into metacercariae (Galaktionov and Bustnes 1995) which may significantly extend the lifespan of the intermediate host (Żbikowska 2011) and increase the chances of the parasite transmission to the final host. The explanation of this phenomenon requires further research.

The last problem presented in our research concerns the possible connection between shell morphs and parasite invasion. In the studied areas, the yellow mid-banded morph of shells (Y 00300) was the most widespread. This result is in line with a recent population study on *Cepaea* spp. from Poland (Ożgo et al. 2019). The detected larvae of *B. mesostoma* were found in similar proportions inside snails with different shell colour. Williams and Rae (2016) underline the lack of dependence between the shell morph and the presence of parasites based on their experiments on the susceptibility of *C. nemoralis* to nematode (*Phasmarhabditis hermaphrodita* (Schneider, 1859)) invasion. On the other hand, although our analysis has not shown a significant relationship between the colour of the snail shell and the presence of parasites, it is worth noting that among the hosts of cercariae only there were more often brightly coloured individuals (Y) than pink (P) and brown (B) ones. It cannot be ruled out that the contrasting yellow-black colour of the snail shell, being a warning signal for predators (Finkbeiner et al. 2014), reduces the likelihood of eating hosts of cercariae unable to grow in a vertebrate.

Our results indicate the need for further research into the life cycle of *B. mesostoma*, not only because of the cognitive value of research on the new natural host species of this parasite, but also because of the threat to poultry farming.

## Electronic supplementary materials

Fig. SM1 Photomicrographs of hepatopancreas of non-infected *Cepaea* sp. (H - Normal hepatopancreatic tubules separated by connective tissues; Tep - normal tubule epithelial cells bound the lesion; N - cell nucleus) (PDF 292 kb)

Table SM1 Set of ITS DNA sequences possessed from Genbank used in phylogenetic reconstruction along with database accession numbers (DOCX 16 kb)
