# Supplementary figures and images for: Cepaea spp. as a source of Brachylaima mesostoma (Digenea: Brachylaimidae) and Brachylecithum sp. (Digenea: Dicrocoeliidae) larvae in Poland
Source: Parasitol Res. 2019 Nov 25;119(1):145–52. doi: 10.1007/s00436-019-06516-2 (PMC6941998; doi:10.1007/s00436-019-06516-2)

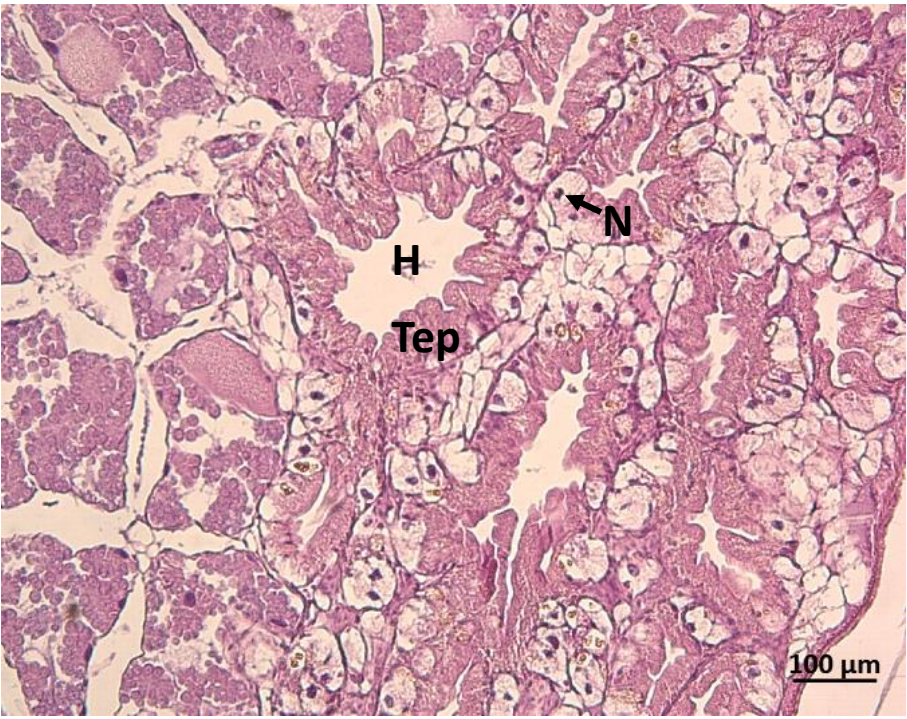

Supplement: Supplementary file 1 — Photomicrographs of hepatopancreas of non-infected Cepaea sp. (H - Normal hepatopancreatic tubules separated by connective tissues; Tep - normal tubule epithelial cells bound the lesion; N - cell nucleus) (PDF 292 kb) [file 436_2019_6516_MOESM1_ESM.pdf]
