# Supplementary material for: Cepaea spp. as a source of Brachylaima mesostoma (Digenea: Brachylaimidae) and Brachylecithum sp. (Digenea: Dicrocoeliidae) larvae in Poland
Source: Parasitol Res. 2019 Nov 25;119(1):145–52. doi: 10.1007/s00436-019-06516-2 (PMC6941998; doi:10.1007/s00436-019-06516-2)
Supplement: Supplementary file 2 — Set of ITS DNA sequences possessed from Genbank used in phylogenetic reconstruction along with database accession numbers (DOCX 16 kb) [file 436_2019_6516_MOESM2_ESM.docx]

Table SM1. Set of ITS DNA sequences possessed from Genbank used in phylogenetic

reconstruction along with database accession numbers

| No. | Species | Accesion number |
| --- | --- | --- |
| 1 | *Alaria mustelae* | JF820609 |
| 2 | *Brachylaima mesostoma* | KT074965 |
| 3 | *Brachylaima mesostoma* | KT074964 |
| 4 | *Brachylaima mesostoma* | KT074967 |
| 5 | *Brachylaima* sp. | JX010634 |
| 6 | *Clinostomoides brieni* | MH238416 |
| 7 | *Clinostomoides brieni* | MH238412 |
| 8 | *Clinostomoides brieni* | MH238415 |
| 9 | *Clinostomoides brieni* | MH238414 |
| 10 | *Clinostomoides brieni* | MH238413 |
| 11 | *Euclinostomum heterostomum* | KP721438 |
| 12 | *Euclinostomum heterostomum* | KP721437 |
| 13 | *Leucochloridium paradoxum* | KP903694 |
| 14 | *Leucochloridium paradoxum* | KP903686 |
| 15 | *Leucochloridium paradoxum* | MH101511 |
| 16 | *Leucochloridium paradoxum* | KP903688 |
| 17 | *Leucochloridium paradoxum* | KP938187 |
| 18 | *Leucochloridium paradoxum* | JN639012 |
| 19 | *Leucochloridium paradoxum* | JF346883 |
| 20 | *Leucochloridium paradoxum* | JF274482 |
| 21 | *Leucochloridium perturbatum* | MK377349 |
| 22 | *Leucochloridium perturbatum* | KP903696 |
| 23 | *Leucochloridium perturbatum* | KP903687 |
| 24 | *Leucochloridium perturbatum* | KP938186 |
| 25 | *Leucochloridium perturbatum* | JN639011 |
| 26 | *Leucochloridium perturbatum* | JF331664 |
| 27 | *Leucochloridium perturbatum* | MK377351 |
| 28 | *Leucochloridium vogtianum* | KP903700 |
| 29 | *Leucochloridium vogtianum* | KP903699 |
| 30 | *Leucochloridium vogtianum* | KP903689 |
| 31 | *Leucochloridium vogtianum* | KP903690 |
| 32 | *Leucochloridium vogtianum* | KP903691 |
| 33 | *Odhneriotrema incommodum* | MF766000 |
| 34 | *Odhneriotrema incommodum* | MF765998 |
